# Sodium excretion is higher in patients with rheumatoid arthritis than in matched controls

**DOI:** 10.1371/journal.pone.0186157

**Published:** 2017-10-13

**Authors:** Sarah Marouen, Guilhem du Cailar, Rachel Audo, Cedric Lukas, Gaelle Vial, Anne Tournadre, Emmanuel Barrat, Jean Ribstein, Bernard Combe, Jacques Morel, Claire I. Daien

**Affiliations:** 1 Rheumatology Department, Lapeyronie Hospital and Montpellier University, Montpellier, France; 2 Internal medicine and hypertension, Lapeyronie Hospital and Montpellier University, Montpellier, France; 3 Institute of molecular genetic, UMR5535, CNRS, Montpellier, France; 4 Rheumatology Department, Gabriel-Montpied Hospital and Clermont-Ferrand University, Clermont-Ferrand, France; 5 Laboratoire LESCUYER, Aytré, France; Mayo Clinic Rochester, UNITED STATES

## Abstract

**Objective:**

It was shown that sodium can promote auto-immunity through the activation of the Th17 pathway. We aimed to compare sodium intake in patients with rheumatoid arthritis (RA) vs. matched controls.

**Methods:**

This case-control study included 24 patients with RA at diagnosis and 24 controls matched by age, gender and body mass index. Sodium intake was evaluated by 24-hr urinary sodium excretion.

**Results:**

Sodium excretion was greater for patients with early RA (2,849±1,350 vs. 2,182±751.7mg/day, p = 0.039) than controls. This difference remained significant after adjustment for smoking and the use of anti-hypertensive and nonsteroidal anti-inflammatory drugs (p = 0.043). Patients with radiographic erosion at the time of diagnosis had a higher sodium excretion than those without (p = 0.028).

**Conclusion:**

Patients with early RA showed increased sodium excretion which may have contributed to autoimmunity.

## Introduction

Rheumatoid arthritis (RA) is an immune disease resulting from an interaction between genetic and environmental factors. Cigarette smoking and bacterial infection (*Porphyromonas gingivalis*) are involved in the development of RA [1,2]. A link between diet (sugar sweetened soda, restrictive carbohydrate nutrients, fish, meat and proteins, and sodium excretion) and the development of RA has been suggested [3,4]. Although data on the impact of diet on RA activity are conflicting, 40% of RA patients still believe that diet has implications for RA [5].

High salt intake may play a role in the development of autoimmune disease [6]. Both *in vitro* and *ex vivo*, excess sodium increases the differentiation and activation of T helper 17 (Th17) cell pathways by inducing serum glucocorticoid kinase 1 (SGK1) [7]. Th17 cells are implicated in the pathogenesis of RA by interacting with macrophages, fibroblast-like synoviocytes, chondrocytes and osteoblasts [8]. Excess sodium may also alter regulatory mechanisms of the innate and adaptive immune system and expand CD14^++^CD16^+^ monocytes, which are also increased in RA [9].

Some clinical studies have suggested that increased sodium excretion based on food questionnaire evaluation was associated with development of RA [4,10], but an association of sodium with activity and severity of RA is still unknown. We aimed to compare sodium excretion using an objective measure in patients with untreated early RA vs. matched controls. The secondary objective was to evaluate the association of sodium excretion with disease activity and severity.

## Methods

### Design

Case-control study.

### Patients

The present study was approved by the medical ethics committee of Montpellier, France (number CPPQ2016.11.01), in accordance with the 1975 Declaration of Helsinki and with article R. 1121–3 of French public health law (April 26, 2006). Written, informed consent was provided by the all patients.

Patients were included from July 2014 to February 2016, in two French regional centres (Montpellier, Limoges). Patients with very early RA were included at the time of the diagnosis. They fulfilled the 2010 ACR/EULAR criteria. They did not receive any treatment for rheumatoid arthritis, including glucocorticoids previous to inclusion.

Controls were healthy subjects or patients consulting in the rheumatology department for mechanical disease, mostly back pain, tendinitis and fibromyalgia. They did not have inflammatory diseases and had not received glucocorticoids in the previous month. Non-inclusion criteria for all patients were infection, active cancer, Addison disease, nephritic syndrome and inappropriate secretion of anti-diuretic hormone. Controls were matched to early RA patients on gender, age ± 5 years, and body mass index (BMI) ± 2 kg/m^2^.

### Radiography

Patients with early RA underwent radiography of hands and feet (anteroposterior and oblique views). Radiographies were read independently by 2 experienced rheumatologists (CD, CL). Erosive status was defined by the presence of at least one erosion.

### Measure of daily salt intake

Sodium intake was evaluated by 24-hr urinary sodium excretion. Urines were collected at the hospital or at home. All patients were informed in detail on how to accurately collect 24-hr urine samples. They were asked to urinate for the first time, flush it down the toilet, and then begin the urine collection. They were asked to collect every drop of urine during the day and night in an empty collection bottle and to finish by collecting the first urine passed the next morning, also in the collection bottle. All patients received the same advice. Patients completed the French validated food frequency questionnaire (FFQ) used to evaluate nutrient intake (e.g., total energy intake, fatty acid, sugar, B12 vitamin), this questionnaire that was previously validated [11]. The aim of FFQ use was to account for confounding alimentary factors.

### Biochemical measures

Rheumatoid factor were measured by immunonephelometry (BN2 siemens); anti-citrullinated protein antibody by chemiluminescence (Bioflash, Werfen), and C-reactive protein by immunoturbidimetry (C8000 roche).

### Data collection

Data collected at inclusion included demographic characteristics: gender, age, weight, height, and BMI; confounding factors for salt intake or urinary excretion of sodium: smoking, hypertension, diuretic treatment, hypothyroid, and cardiac failure; and disease characteristics: disease duration, presence of rheumatoid factor (RF), and/or anti-citrullinated protein antibody (ACPA), presence or absence of radiographic erosions, rheumatoid nodule, activity evaluated by Disease Activity in 28 joints-C-reactive protein level (DAS28-CRP) and CRP level at inclusion (mg/L).

### Statistical analysis

The characteristics of patients were described with proportions for categorical variables and mean ±SD or median (interquartile range [IQR]) for continuous variables. The distribution of the latter was tested by the Shapiro-Wilk statistic. Comparisons of 2 groups involved Student t test when data distribution was normal; otherwise, Mann-Whitney sum rank tests were used. Correlations between continuous parameters were evaluated by Spearman’s rank correlation coefficient. Two-tailed P<0.05 was considered statistically significant. Analysis of covariance (ANCOVA) was used to adjust the difference of sodium excretion between RA and controls for use of anti-hypertensive drugs, nonsteroidal anti-inflammatory drugs (NSAID) and smoking status. Partial eta squared was used to evaluated the effect size.

24-hr urinary sodium excretion was estimated at 2,533±788 mg/day, on average, in French women [12]. We hypothesized a one-standard deviation difference between patients with early RA and matched controls, thus a difference of 788 mg/day. This choice is clinically relevant because reducing the mean sodium consumption by 400 mg in the population has been projected to prevent up to 28,000 deaths from any cause [13]. With an alpha risk of 5% and a power of 90%, the number of patients needed to demonstrate increased consumption of salt in those with early RA was 22 patients per group. All raw data are available on S1 Dataset.

## Results

### Patient characteristics

48 patients were included: 24 with early RA and 24 matched controls. Patient characteristics are in Table 1. The details of NSAID and anti-hypertensive drugs are available in S1 Table.

**Table 1:** Baseline characteristics of patients with RA at diagnosis (early RA) and matched controls.

	Control	Early RA
**Patients**, no	24	24
**Women** no. (%)	17 (71)	17 (71)
**Age, mean (years)**	58±13	58±14.3
**BMI, mean (kg/m**^**2**^**)**	24.5±3.6	24.0±3.7
**Smokers** no. (%)		
***Current***	5 (21)	5 (21)
***Past***	1 (4)	8 (33)
***Never***	18 (75)	11 (46)
**Use of anti-hypertensive drugs**, no. (%)	6 (25)	6 (25)
**Diuretics**, no. (%)	0	4 (16.6)
**Hypothyroidism**, no. (%)	3 (12.5)	3 (12.5)
**Renal failure**, no. (%)	0	0
**Cardiac failure**, no. (%)	0	0
**Radiographic erosion**, no. (%)	-	7 (29)
**Time from diagnosis, mean (years)**	-	0
**RF+**, no. (%)	-	14 (58.3)
**ACPA+**, no. (%)	-	14 (58.3)
**CRP, median (IQR) (mg/L)**	-	17 [6.1–54.2]
**DAS28-CRP, median (IQR)**	-	4.1 (3.5–4.6)
**DAS28**-**CRP > 3.2**, no. (%)		19 (79.2)
**Rheumatoid nodules**, no. (%)	-	0
**Glucocorticoids**, no. (%)	-	0
**NSAID**, no. (%)	8 (33.3)	10 (41.7)
**Synthetic DMARD**, no. (%)	-	0

DMARD: Disease Modifying Anti Rheumatic Drug; NA: Non Applicable; Nonsteroidal anti-inflammatory drugs; RF+: rheumatoid factor positivity(measured by immunonephelometry, RF>15 UI/ml); ACPA+: anti-citrullinated protein antibody positivity (measured by chemiluminescence, ACPA>4.6 UI/ml); CRP: C-reactive protein (measured by immunoturbidimetry, N<5 mg/l); DAS28: Disease Activity in 28 Joints

### Sodium excretion greater in patients with RA than controls

**Fig 1:**
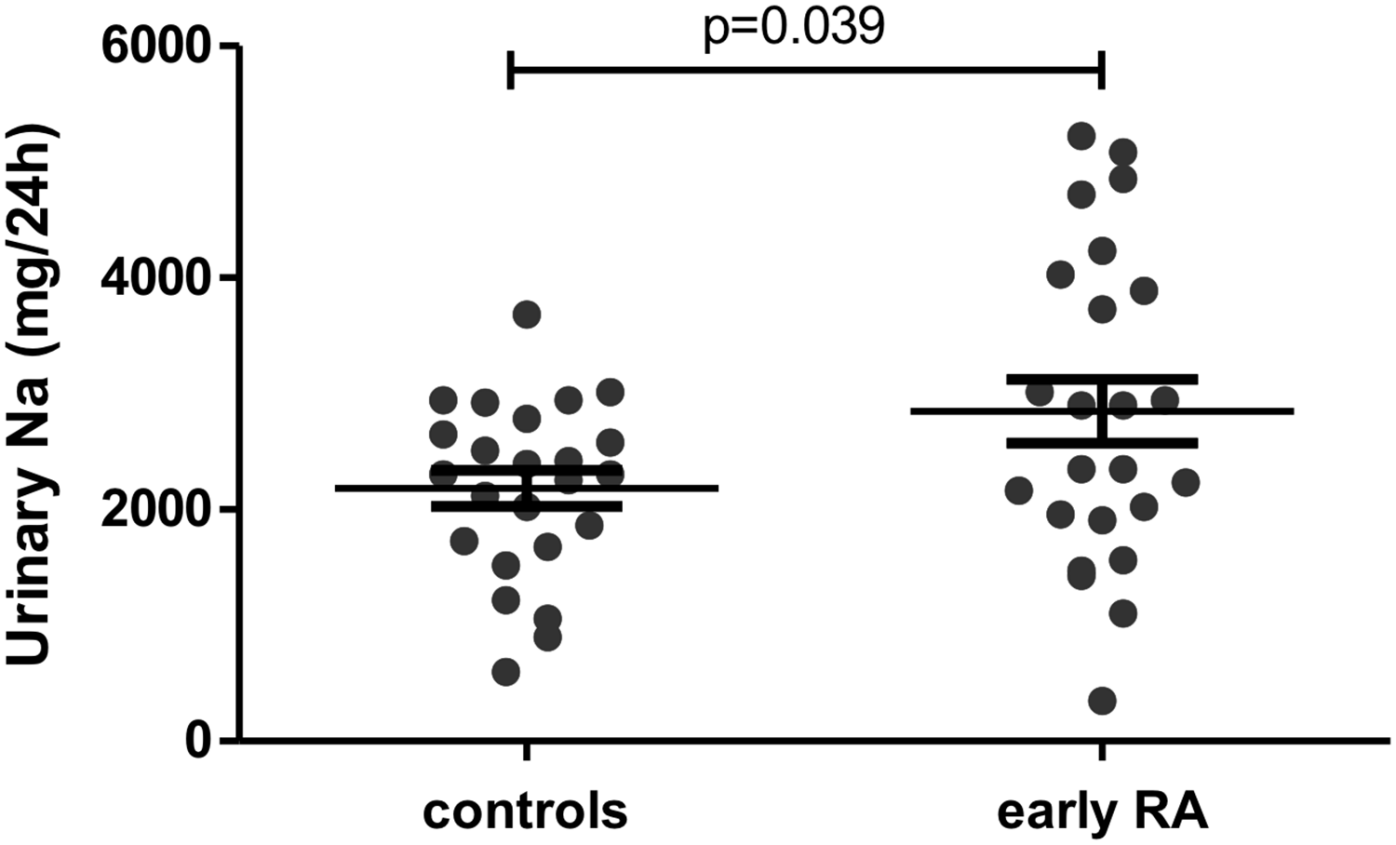
Sodium excretion was higher in patients with early RA than in matched controls. Sodium intake was assessed by 24-hr urinary sodium excretion. Horizontal bars are means and whiskers are SEM. Student *t* test was used.

Sodium excretion was significantly higher in patients with early RA than matched controls (2,849±1,350 vs. 2,182±751.7 mg/day, p = 0.039) (Fig 1). This difference remained significant after adjustment for the use of hypertensive drugs, NSAID and smoking (ANCOVA, p = 0.043) (Table 2).

**Table 2:** Multi-adjusted analysis of sodium excretion in early RA and matched controls.

Dependent variable: 24-hour sodium excretion	partial eta squared	p
Corrected model	0.223	0.031
Smoking (yes/no)	0.023	0.337
Anti-hypertensive drugs (yes/no)	0.115	0.026
Nonsteroidal anti-inflammatory drugs (yes/no)	0.044	0.179
Disease (rheumatoid arthritis yes/no)	0.096	0.043

ANCOVA analysis adjusted for smoking, anti-hypertensive and nonsteroidal anti-inflammatory drug use.

The sodium intake estimated by FFQ significantly, albeit weakly, correlated with 24-hour sodium excretion (R = 0.152, p = 0.026). It was numerically higher in patients with early RA but not significantly (2,123±1,034 vs. 2,050±603 mg/day respectively for RA and controls; p = 0.77). Nutrient intake evaluated by the FFQ did not differ, except for B12 vitamin, whose intake was higher for patients with early RA than matched controls (5.57 vs. 3.81 mg/day, p = 0.036) (Table 3).

**Table 3:** Daily nutrient intake in patients with early RA and matched controls.

	Early RA	Controls	P value
Total energy (kcal)*	1,886	1,775	0.60
**Proteins (g)**	73	66	0.36
**Lipids (g)**	90	80	0.36
**Carbohydrate (g)**	170	183	0.55
**Simple sugar (g)**	50	58	0.37
**Fibres (g)**	20	23	0.32
**Fatty acids (g)**	30	27	0.55
**MUFA (g)**	40	32	0.17
**PUFA (g)**	14	15	0.91
**Vitamin A (g)**	530	402	0.24
**Vitamin D (g)**	4	2.5	0.13
**Vitamin E (mg)**	4.6	3.1	0.14
**Vitamin C (mg)**	139	169	0.28
**Vitamin B1 (mg)**	1.1	1.1	0.64
**Vitamin B2 (mg)**	1.6	1.6	0.66
**Vitamin B3 (mg)**	15.6	14.1	0.31
**Vitamin B5 (mg)**	5.2	5.1	0.81
**Vitamin B6 (mg)**	1.7	1.6	0.67
**Vitamin B9 (mg)**	336	347	0.78
**Vitamin B12 (mg)**	5.67	3.81	0.04
**Magnesium (mg)**	272	274	0.93
**Calcium (mg)**	690	677	0.90
**Phosphorus (mg)**	1,165	1,103	0.63
**Potassium (mg)**	2,868	2,918	0.89
**Iron (mg)**	12.7	12	0.53
**Zinc (mg)**	9	8.2	0.35
**Copper (mg)**	1.4	1.3	0.71
**Manganese (mg)**	2.9	3.2	0.34
**Iodide (g)**	111	95	0.32
**Selenium (g)**	49	42	0.18
**Cholesterol (mg)**	269	230	0.23

* Total energy calculation takes into account an estimate of alcohol intake. However, as the questionnaire is not validated for alcohol consumption due to declarative biases, this data is not presented. MUFA: monounsaturated fatty acids; PUFA: polyunsaturated fatty acids.

### Sodium excretion and disease parameters

**Fig 2:**
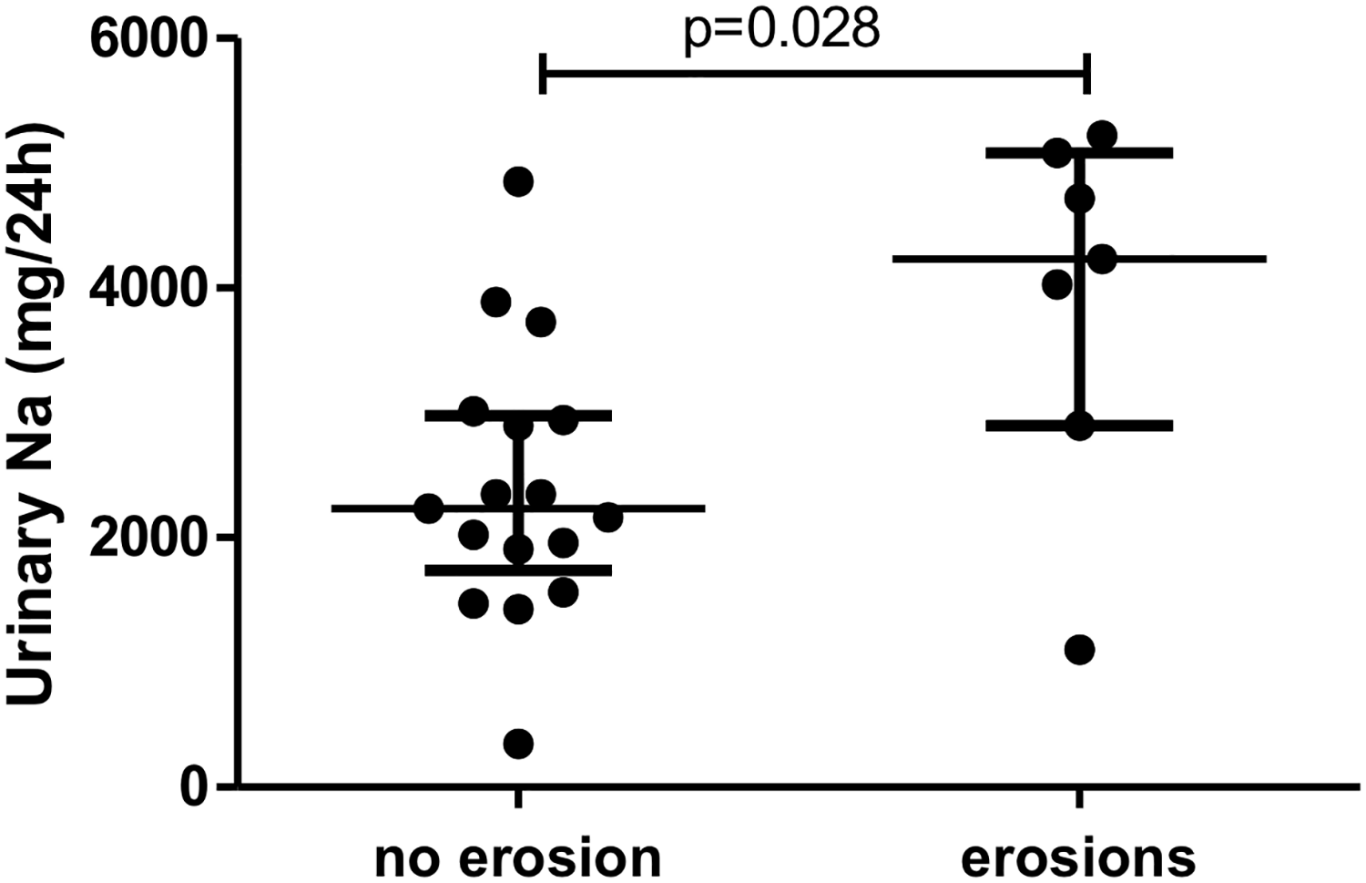
Sodium excretion was higher in early RA patients with erosions than in those without. Horizontal bars are median and whiskers are IQR compared by Mann-Whitney test.

24-hr urinary sodium excretion was greater for patients with early RA and with (n = 7) than without (n = 17) radiographic erosion at diagnosis (median [IQR] 4,232 [2,898–5,083] vs. 2,231 [1,737–2,979] mg/d sodium, p = 0.028) (Fig 2).

In patients with early RA, patients with and without auto-antibodies did not differ in sodium excretion (2,645 [1,995–3,973] vs. 2,622 [1,530–4,198] for RF+ vs. RF-, respectively; p = 0.725). 24-hr urinary sodium excretion was not correlated with disease activity evaluated by DAS28-CRP (r^2^ = 0.00, p = 0.93) (S1 Fig) or CRP level (r = -0.28, p = 0.19) in patients with early RA.

## Discussion

Sodium excretion, assessed by 24-hr urinary sodium excretion, was significantly higher in patients with early RA (at diagnosis) than in controls after adjustment for the main confounding factors. Sodium excretion was greater in those with erosions than without. Sodium excretion was not correlated with RA activity.

Two recent studies assessed sodium consumption in RA patients [4,10]. The first found an association between high sodium consumption among smokers and risk of RA development in patients who assessed their diet habits 7 years before RA development. The second showed a dose-dependent relationship between daily sodium intake and patient-reported diagnosis of RA. Both studies evaluated sodium intake by FFQ only.

High salt intake may trigger inflammation in pre-clinical RA via its effects on immune cells. In animal models and human cells, sodium excess may induce differentiation and activation of Th17 cells by inducing SGK1 [7]. *In vitro*, sodium was associated with T-cell secretion of interleukin 17 (IL-17) and tumor necrosis factor α (TNF-α) [7]. IL-17 is involved in bone loss by inducing the RANK/RANKL system [14]. Sodium may expand CD14^++^CD16^+^ monocytes and alter regulatory mechanisms of the innate and adaptive immune systems by causing a functional deficit of M2 macrophages and FOXP3^+^ regulatory T cells [9]. A recent study showed that a low salt diet ameliorated collagen-induced arthritis [15].

Our study does present some limitations. The number of included patients was limited and so the results will need to be confirmed in an independent cohort. Moreover, to demonstrate that high sodium excretion is a risk factor for the development of RA, large prospective studies should be conducted with a long-term follow-up to assess incident RA. All patients were seen in a hospital, which led to a selection bias. Indeed, 7/24 (29%) of those with early RA showed erosive disease, which is higher than the literature data [16]. Another limitation is that sodium consumption was assessed at diagnosis and we cannot exclude that patients have modified their diet after symptom onset. Finally, whilst 24-hour sodium excretion is the gold-standard to assess sodium consumption in the general population, variability can be observed. Repeated measurements of 24-hour sodium excretion might have improved precision [17]. However, this would have been difficult to conduct in patients and could have been influenced by treatment with disease-modifying anti-rheumatic drugs after diagnosis.

No study has clearly evaluated the influence of rheumatoid arthritis on sodium excretion. Natriuresis may be impacted by inflammatory states only in acute situations. In equilibrate state, when renal and left ventricular function are preserved and according to “pressure-natriuresis” regulatory system, long-term salt balance is not impacted. Thus, we believe that natriuresis is also reliable to evaluate sodium intake in RA patients.

The FFQ was used to control potential biases from nutrient intake. No difference was observed for intake of other nutrients, apart from Vitamin B12 which comes mainly from animal sources. However, we cannot exclude that the observed difference in sodium excretion could reflect other dietary differences.

The strengths of our study include a gold standard measurement of sodium excretion by 24-hr urinary sodium excretion. Patients were carefully characterized by a double-blind assessment of erosions. Our study also evaluated the impact of salt intake on RA activity and severity.

In conclusion, this study found a higher sodium excretion in patients with RA compared to controls, with potential association on RA severity. Further longitudinal studies are required. The impact of low-sodium diet in patients with RA should be explored.

## Significance and innovations

- Salt excretion is higher in patients with early RA than in matched controls
- Patients with radiographic erosions at diagnosis have higher salt excretion than patients without

## Supporting information

S1 Dataset Raw data. (XLS)

S1 Fig Correlation between Disease Activity in 28 joints (DAS28) and 24-hr sodium excretion in patients with early RA. (DOCX)

S1 Table Anti-hypertensive drugs used in patients and controls. (DOCX)
