# Supplementary material for: Sodium excretion is higher in patients with rheumatoid arthritis than in matched controls
Source: PLoS One. 2017 Oct 13;12(10):e0186157. doi: 10.1371/journal.pone.0186157 (PMC5640209; doi:10.1371/journal.pone.0186157)
Supplement: S1 Fig — (DOCX) [file pone.0186157.s002.docx]

**Supplementary Figure 1. Correlation between Disease Activity in 28 joints (DAS28) and 24-hr sodium excretion in patients with early RA.**

Spearman’s rank was used.
