# Supplementary material for: Sodium excretion is higher in patients with rheumatoid arthritis than in matched controls
Source: PLoS One. 2017 Oct 13;12(10):e0186157. doi: 10.1371/journal.pone.0186157 (PMC5640209; doi:10.1371/journal.pone.0186157)
Supplement: S1 Table — (DOCX) [file pone.0186157.s003.docx]

**Supplementary Table 1. Anti-hypertensive drugs used in patients and controls.**

| **Anti-hypertensive drugs** | **Control** | **Early RA** |
| --- | --- | --- |
| **N** | 6 | 6 |
| **Diuretics** | 0 | 1 |
| **Calcium channel blockers** | 1 | 2 |
| **Angiotensin II receptor antagonists** | 3* | 2 |
| **Beta blockers** | 3* | 1 |

*One patient treated with an **Angiotensin II receptor antagonist and a beta blocker**
